# Response of the Phytoplankton Sinking Rate to Community Structure and Environmental Factors in the Eastern Indian Ocean

**DOI:** 10.3390/plants11121534

**Published:** 2022-06-08

**Authors:** Xingzhou Wang, Jun Sun, Yuqiu Wei, Xi Wu

**Affiliations:** 1 College of Food Science and Engineering, Tianjin University of Science and Technology, Tianjin 300457, China; wxz445079509@163.com; 2 Research Centre for Indian Ocean Ecosystem, Tianjin University of Science and Technology, Tianjin 300457, China; 3 College of Marine Science and Technology, China University of Geosciences (Wuhan), Wuhan 430074, China; wuxi1201@foxmail.com; 4 State Key Laboratory of Biogeology and Environmental Geology, China University of Geosciences (Wuhan), Wuhan 430074, China; 5 Yellow Sea Fisheries Research Institute, Chinese Academy of Fishery Sciences, Qingdao 266071, China; weiyuqiu@163.com

**Keywords:** phytoplankton sinking rate, community structure, eastern indian ocean, environmental factors, oligotrophic

## Abstract

The phytoplankton sinking rate in the eastern Indian Ocean was measured during spring 2017 based on the SETCOL method. The range of phytoplankton sinking rates was −0.291 to 2.188 md^−1^, with an average of 0.420 ± 0.646 md^−1^. The phytoplankton sinking rate in the Equator (EQ) and the eastern boundary of the Indian Ocean near Sumatra (EB) was lower than that in the Bay of Bengal (BOB). The sinking rate above 100 m was low and increased rapidly below 100 m in all the three regions. The phytoplankton community composition had an important impact on the phytoplankton sinking rate in the east Indian Ocean. The strong stratification in BOB resulted in *Trichodesmium* spp. bloom and a lower phytoplankton diversity and evenness in BOB, while the phytoplankton in the deep layer are senescent cells that sink down from the upper layer and cannot actively regulate the state of the cells, resulting in a higher sinking rate. Depth and temperature have a great impact on the physiological state of phytoplankton. The sinking rate of phytoplankton depend on the dominant groups composing the phytoplankton community. For the eastern Indian Ocean, seawater stratification caused by temperature changes the distribution of nutrients in the upper layer, and phytoplankton are affected by temperature and nutrients, resulting in changes in community structure, and finally showing different subsidence characteristics.

## 1. Introduction

As important primary producers in marine ecosystems, phytoplankton are widely distributed and play significant roles in regulating the marine ecosystem and global carbon cycle [1]. Rapidly sinking phytoplankton cells are the main contributors to marine carbon sink [2]. Phytoplankton are affected by gravity while moving with ocean currents in seawater; many studies have shown that the impact of water flow on phytoplankton is also extremely important. Scientists believed that water disturbance could reduce the precipitation rate of phytoplankton [3,4]. Other scientists have suggested that water disturbance accelerates the rate of phytoplankton deposition [5]. Phytoplankton also change their density to obtain the best living condition. It is now clear that phytoplankton cells can regulate their sinking rates. in various ways. For example, dinoflagellates can move autonomously to maintain an optimal depth in the sea; diatoms can selectively absorb some ions or stored fats to change cell density [6,7,8]; illumination changes the growth rate of phytoplankton by affecting their enzymatic reactions [9]. The relationship between environmental factors and phytoplankton communities is an important factor in determining phytoplankton settlement. Therefore, it is not enough to determine the sinking rate of phytoplankton in an entire sea by a single factor. More detailed studies on phytoplankton’s sinking rate should examine other factors, such as phytoplankton cell density, cell physiological activity measurement, and sea water characteristics. Some environmental factors, such as nutrient concentration, irradiance, water viscosity, and turbulence, also indirectly affect the sinking rates [8,10]. 

Stokes’s [11] Law is used to calculate the sinking rate of simple particles. The sinking rates of organic particles in seawater depend on the properties of organic particles and seawater. However, phytoplankton have different shapes and sizes, and each cell has a different physiological state. Studies show that Stokes’s Law is not always valid for phytoplankton cells. To accurately measure the sinking rate of phytoplankton cells, more methods have been proposed—for example, observation under a microscope [12,13,14] and fluorescence detection [15,16,17]. However, these methods can not be widely applied to the actual investigation. The SETCOL method was established by Bienfang [18]. The sinking rates of phytoplankton cells in the corresponding layer can be calculated in the specially designed column by observing and calculating the abundance of phytoplankton cells in different areas. In addition, the sinking rates of a single species can be calculated by microscopy. This method is easy to operate, and more parallel samples can be measured to further reduce systematic error. Furthermore, the sinking details of phytoplankton and particulate information are also collected simultaneously [19,20,21,22]. 

As part of the global ocean cycle, the eastern Indian Ocean (EIO) is bounded by the Indian subcontinent to the north, the Arabian Sea to the west, and the Indonesian islands to the east. EIO is an important warm pool [23]. Influenced by the topography, the interaction between the cold air from the Himalayas and the warm and humid air flow from the Indian Ocean makes this area form an obvious monsoon climate. In the spring monsoon period, the vertical mixing in this area is weak [24], which makes the mixing layer in this area move upward and the available nutrients for phytoplankton decrease [25]. Compared with the Pacific and Atlantic oceans, the study of hydrodynamics and biology is scarce in the Indian Ocean [26]. The phytoplankton sinking rate is an important component of the global ocean carbon cycle, so it is necessary to explore the relationship between the phytoplankton sinking rate and environmental factors. At present, the SETCOL method described by Bienfang [18] is generally accepted as the most accurate method for calculating the precipitation rate of phytoplankton, which can measure not only the phytoplankton community sinking rate but also the species-specific sinking rate. A recent study has shown that in the Changjiang (Yangtze River) estuary, a significant correlation was observed between phytoplankton sinking rate and phytoplankton community structures in the surface layer: during the bloom of some Bacillariophyta the sinking rate resulted higher than during bloom of some dinoflagellates [27]. The SETCOL method was used to study the sinking rates of phytoplankton cells in the EIO to explore various factors affecting the sinking rates of phytoplankton, and to provide some insights to estimate the carbon sink in this area.

## 2. Methods

### 2.1. Study Area and Sampling Stations

**Figure 1:**
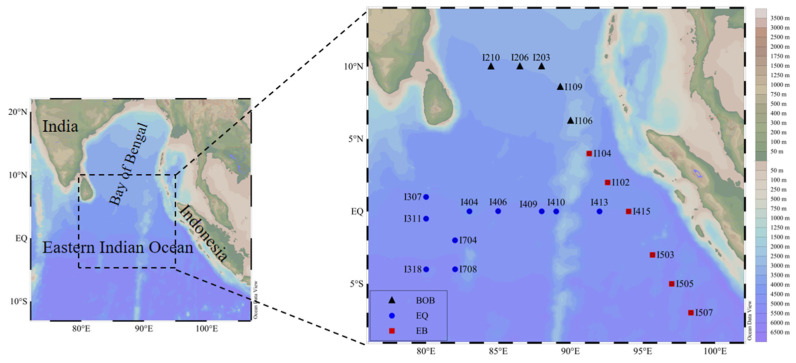
Maps of the study area and the distribution of sampling stations (BOB: Bay of Bengal; EQ: Equator; EB: Eastern Boundary).

This cruise was conducted on the R/V Shiyan Ⅲ during spring 2017 (27 February–22 April). At each station, seawater samples were collected from seven depths within the upper 200 m water column at 21 stations (Figure 1) using a Rosette sample system equipped with a SeaBird CTD (conductivity, temperature, and depth; SBE 19 Plus). Temperature, salinity, and depth were simultaneously recorded in situ. 

### 2.2. Sampling and Analysis

Phytoplankton sinking rates were selected in four layers, including surface (3 m), chlorophyll *a* (Chl-*a*) maximum layer (75 m), middle layer (100 m), and bottom layer (200 m). Six stations (I102, I106, I311, I409, I505, and I708) were selected to measure the sinking rates of the seven layers for more information above 200 m. The SETCOL method reported by Bienfang [28] was used to measure the sinking rates. A plexiglass column with a height of 0.48 m and a volume of 1040 mL was filled with homogenous seawater completely and capped. The plexiglass column was placed in an undisturbed, dark environment for 2–3 h. The experiment was terminated by draining the plexiglass compartment of upper, middle, and bottom layers successively through the wall. Combining the phytoplankton biomass in three compartments with the initial biomass, the sinking rate was calculated according to the formula [18]:

$$\psi =(B_{s}/B_{t})\times L/t.$$

Here, *ψ* is the sinking rate; *B_s_* is the biomass settled into the bottom compartment; *B_t_* is the total biomass in the column; *L* is length of column; *t* is settling interval. For each sampling station, four repeated settling columns were filled with water collected from each sampling depth. Three water samples were collected for Chl-*a* measurement to determine the sinking rate of the phytoplankton community. Water samples were collected from the remaining columns for phytoplankton taxonomic analysis, which was used to determine species-specific sink rates.

The nutrient water sample was pre-filtered through a 0.45 µm cellulose acetate membrane filter and then refrigerated at −20 ℃ for further analysis. Nutrient concentrations including ammonium, phosphate, nitrate, and silicate were examined by Technicon AA3 Auto-Analyzer (Bran + Luebbe). The concentrations of nitrate and ammonium were determined by copper-cadmium column reduction method and indiophenol blue spectrophotometry respectively. Dissolved inorganic silica (DSi) and phosphorus (DIP) were measured using typical spectrophotometric methods [29,30,31]. 

The concentration of Chl-*a* was determined using the extraction fluorescence method. One liter of seawater was filtered through GF/F filters (0.7 μm porosity). The filter was placed into a 10 mL brown glass tube, then 5 mL of acetone with a volume fraction of 90% was added into the glass tube and stored in the dark at 4 °C for 24 h. The Chl-*a* fluorescence was measured in non-acidified mode using the Turner Fluorometer (model 10-AU) and calculated according to the formula by [32]. 

The water samples for phytoplankton analysis were stored with 2% (final concentration) buffered formalin. Phytoplankton species were identified according to [33] and [34] using an improved Utermöhl method under an inverted microscope (Motic AE 2000).

The abundance of three Pico groups were quantified by FCM (Becton–Dickinson Accuri C6) according to the study of Troussellier [35]. A volume of 1.5 mL of seawater was taken at the corresponding depth at the sinking station, and 0.5 mL paraformaldehyde was added. Then, only 196 μL samples were used for analysis at a speed of 66 μL/min for 3 min. The sample injection chamber volume allows for a direct cell count per μL.

### 2.3. Data Analysis

The Shannon–Wiener diversity index (*H’*) was used to calculate the species diversity index [36]. The Pielou index (*J*) was used to calculate the species evenness index (*J*) [37]. The abundance of phytoplankton cells in water column is calculated by trapezoidal integral method [38]:

$$P=\left\{\sum_{i=1}^{n-1}\frac{P_{i+1}+P_{i}}{2}\left(D_{i+1}-D_{i}\right)\right\}/D$$

where *P* is the average of phytoplankton abundance in water column, *P_i_* is the abundance value of phytoplankton in layer *i*, *D* is the maximum sampling depth, *D_i_* is the depth of layer *i*, and *N* is the sampling level.

The relationship between sinking rate and community structure and environmental factors was analyzed using SPSS 19.0 (Pearson correlation test). Cluster analysis (Primer 6.0) was used for the similarity analysis of the phytoplankton community structure. An aggregated boosted tree (ABT) analysis was performed to quantify the effect of the community structure and environmental factors on the sinking rate using the “gbmplus” package with 500 trees for boosting in R. We constructed generalized additive models (GAMs) using the R package “mgcv” to fit the responses of sinking rate to community structure and environmental factors. Figures depicting horizontal and vertical phytoplankton distributions were constructed using Ocean Data View 4.10, Origin 8.5, Arc GIS 10.2., and R 4.1.0.

## 3. Results

### 3.1. Hydrographic Conditions

**Figure 2:**
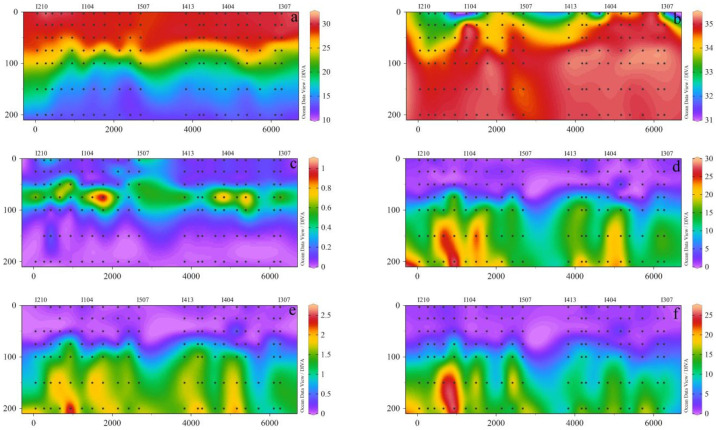
The vertical distributions of (**a**) temperature (°C), (**b**) Salinity (psu), (**c**) Chlorophyll a (μg/L), (**d**) DIN (μmol/L), (**e**) DIP (μmol/L), (**f**) DSi (μmol/L) in the eastern Indian Ocean in spring 2017. The bottom x axis was a straight line distance between two stations, and the top x axis was the station. Dissolved Inorganic Nitrogen (DIN), Dissolved Inorganic Phosphorus (DIP), Dissolved Silicate (DSI).

The ranges of surface temperature, salinity, and Chl-*a* concentrations were 28.53–31.07 ℃ (average = 29.70 ± 0.55 ℃), 31.16–34.71 (average = 33.13 ± 1.05), and 0.009–1.028 μg L^−1^ (average = 0.206 ± 0.209 μg L^−1^), respectively. In the vertical distribution, the temperature decreased with depth, and salinity increased from the surface to the bottom (Figure 2a,b). The concentrations of Chl-*a* increased and then decreased with depth (Figure 2c), and the maximum value occurred at around 75 m. High surface temperature and salinity were particularly observed near the equator between 80 and 90° E due to the apparent influence of the Wyrtki jets (WJ). The concentrations of nutrients in the EIO were relatively low in EIO. The concentrations of nutrients in BOB and EB were significantly higher than in EQ (Figure 2d–f), indicating that the nutrients may be supplemented by the marginal sea, which usually serves as a nutrient sink. Overall, the environmental factors of BOB were different from those of the EQ and EB. The nutrients of BOB were affected by upwelling and the input of nearshore nutrients, and the concentration of nutrients was significantly higher in BOB than in the other two regions.

### 3.2. Phytoplankton Community Structure

A total of 342 species (73 genera and 4 phyla) of phytoplankton were identified across the EIO. A total of 249 dinoflagellates species were accurately discriminated, representing 72.81% of the total phytoplankton taxa. Bacillariophyta were the second most diverse group (85 species), accounting for 24.85% of the total species. Cyanophyta were the third most diverse group (4 species), accounting for 1.17% of the total species. The dominant taxa in the BOB belonged to Bacillariophyta (four species), Dinoflagellates (four species), Cyanobacteria (one species), and Chrysophyte (one species) (Table 1). In particular, Trichodesmium spp. (0–2.9 × 10^5^ cells/L) became the most common dominant species and were mainly distributed in the water layer above 75 m.

**Figure 3:**
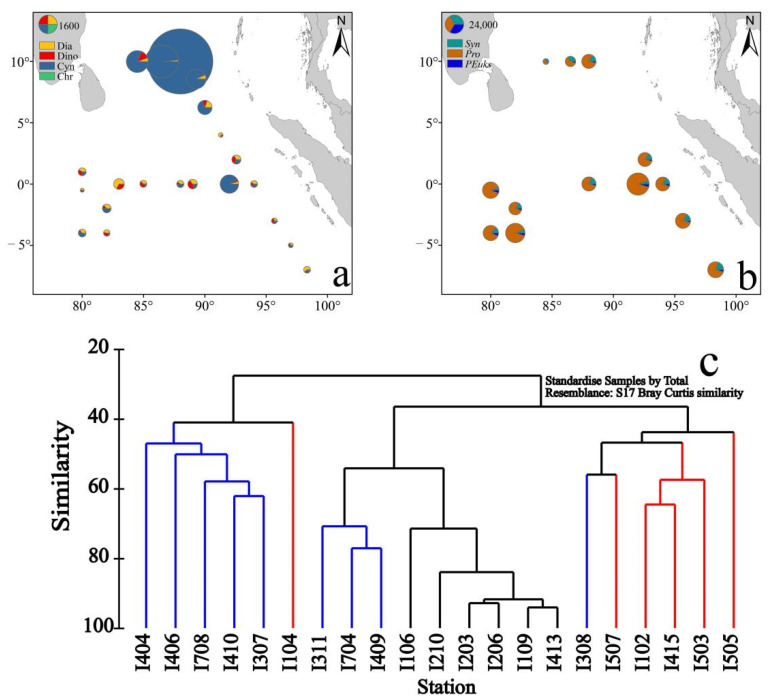
Horizontal distribution and cluster analysis of phytoplankton. (**a**) Horizontal distribution of Microphytoplankton (cells/L) (Diatoms: Dia; Dinoflagellate: Dino; Cyanobacteria: Cyn; Chrysophyta: Chr), (**b**) Horizontal distribution of Picophytoplankton (cells/mL) (weighted average) (*Synechococcus: Syn; Prochlorococcus: Pro; Picoeukaryotes: PEuks*), (**c**) Cluster analysis of phytoplankton community structure (weighted average). The blue line indicates the station name of EQ, the red line indicates the stations of EB, and the black line indicates the station name of BOB.

The abundance of different phytoplankton species presented various distributions in the EIO. The cyanobacteria abundance was the highest, with a mean abundance of (8819.36 ± 4452.67) cells/L. The mean abundance of diatoms and dinoflagellates was 113.00 ± 192.52 and 96.54 ± 153.72 cells/L, respectively. Chrysophyceae was the lowest, with an average abundance of 14.70 ± 19.00 cells/L. According to the average abundance of phytoplankton at each station, the phytoplankton abundance was dominated by cyanobacteria in the BOB, among which the highest abundance of cyanobacteria was at station I206. The abundance of diatoms and dinoflagellates are similar (Figure 3a). *Prochlorococcus* (*Pro*) presented an obvious abundance advantage in the EIO for picophytoplankton (Figure 3b). The cell abundance of *Pro* (12347.47 ± 21183.40 cells/mL) was the highest, the abundance of *Synechococcus* (*Syn*) was 2457.97 ± 1818.23 cells/mL, and the abundance of *Picoeukaryotes* (*PEuks*) was 777.93 ± 1088.13 cells/mL (Figure 3b). Cluster analysis was carried out on account of the cell abundance value and species number of each station (Figure 3c). According to the Bray–Curtis similarity, the survey area was divided into three groups (i.e., BOB, EB, and EQ). The cluster analysis results were similar to the geographical distribution of the survey area.

**Figure 4:**
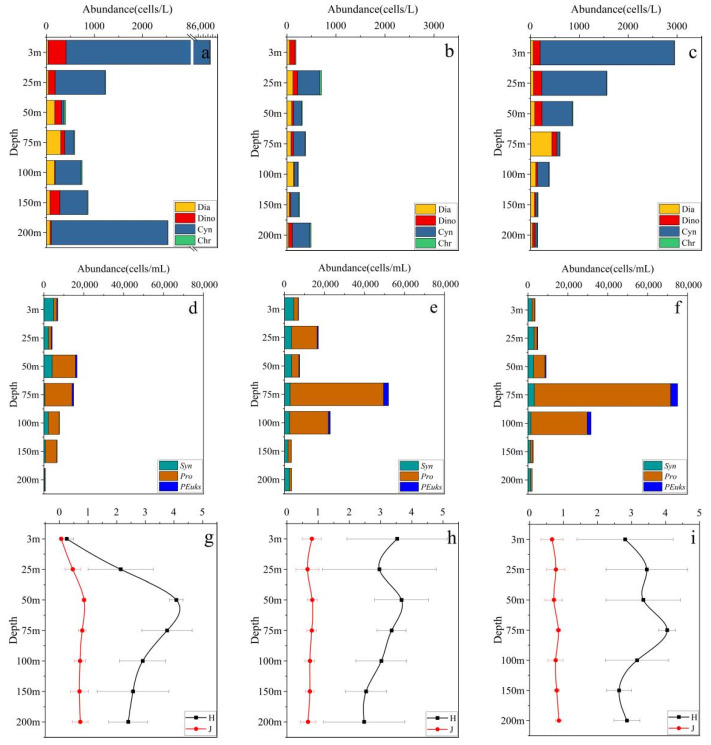
Vertical distribution of Microphytoplankton (Diatoms: Dia; Dinoflagellate: Dino; Cyanobacteria: Cyn; Chrysophyta: Chr), Picophytoplankton (*Synechococcus: Syn; Prochlorococcus: Pro; Picoeukaryotes:* *PEuks*), Shannon-Wiener diversity index (*H’*), and Pielou index (*J*) in different sub-regions of the eastern Indian Ocean in spring 2017. (**a**) Microphytoplankton in BOB, (**b**) Microphytoplankton in EB, (**c**) Microphytoplankton in EQ, (**d**) Picophytoplankton in BOB, (**e**) Picophytoplankton in EB, (**f**) Picophytoplankton in EQ, (**g**) *H’* and *J* in BOB, (**h**) *H’* and *J* in EB, (**i**) *H’* and J in EQ.

The vertical distributions of phytoplankton were different in these three regions. The microphytoplankton in the three regions are mainly composed of diatoms, dinoflagellates, and *Trichodesmium* sp. The diatoms in the three regions increased in the upper 75 m and then gradually decreased with depth. Dinoflagellates decreased from the surface to the bottom. Cyanobacteria are mainly distributed in the upper layer. The abundance of *Trichodesmium* sp. in the BOB was higher than in the other two regions. The vertical distributions of picophytoplankton were similar in the three regions, while the abundance of picophytoplankton was the lowest in the BOB (Figure 4a–c). For *Syn*, the maximum abundance was observed in the surface layer (3–50 m) and then gradually decreased with increasing depth. In contrast, the abundance of *Pro* reached the maximum at depths of 50–75 m and then rapidly dropped to a minimum in the bottom layer (150–200 m). *PEuks* had similar vertical distributions with *Pro* but was 1–2 orders of magnitude lower than *Pro* (Figure 4d–f). Comparing the Shannon–Wiener diversity index (*H’*) of the three regions, the Shannon–Wiener diversity index *(H’*) of BOB is the lowest at the upper layers, and the Shannon–Wiener diversity index (*H’*) of EB and EQ are at the bottom layers (Figure 4g–i). The trend of the Pielou index *(J*) is similar to that of the Shannon–Wiener diversity index (Figure 4g–i).

### 3.3. Sinking Rates

**Figure 5:**
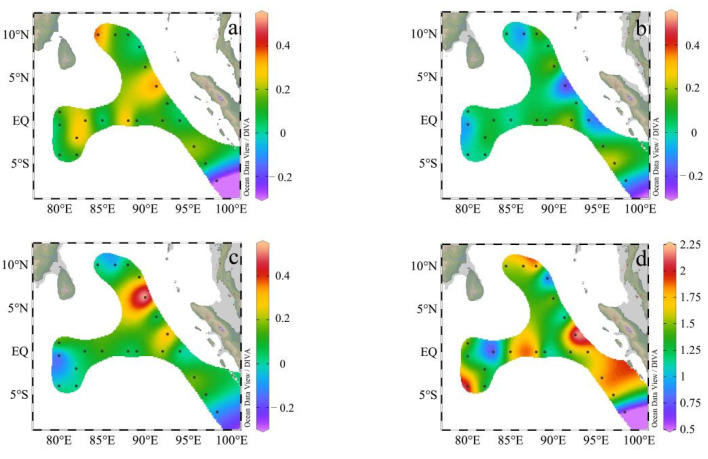
Distribution of sinking rates in the eastern Indian Ocean in spring 2017. (**a**) Horizontal distribution of sinking rates in 3 m. (**b**) Horizontal distribution of sinking rates in 75 m. (**c**) Horizontal distribution of sinking rates in 100 m. (**d**) Horizontal distribution of sinking rates in 200 m.

The average sinking rates in the EIO showed different distributions (Figure 5), with a range of −0.291–2.188 md^−1^ (average = 0.420 ± 0.646 md^−1^). Comparing the sinking rates in different depths (Figure 5), the sinking rates of phytoplankton were similar and relatively low above 100 m, and then the sinking rates increased rapidly below 100 m. The horizontal sinking rates in the four layers of 5 m, 75 m, 100 m, and 200 m were compared in the survey area. It was found that the sinking rates of each layer were not uniform, and the sinking rates in 200 m in the EB were higher than in the other two regions (Figure 5c,d).

**Figure 6:**
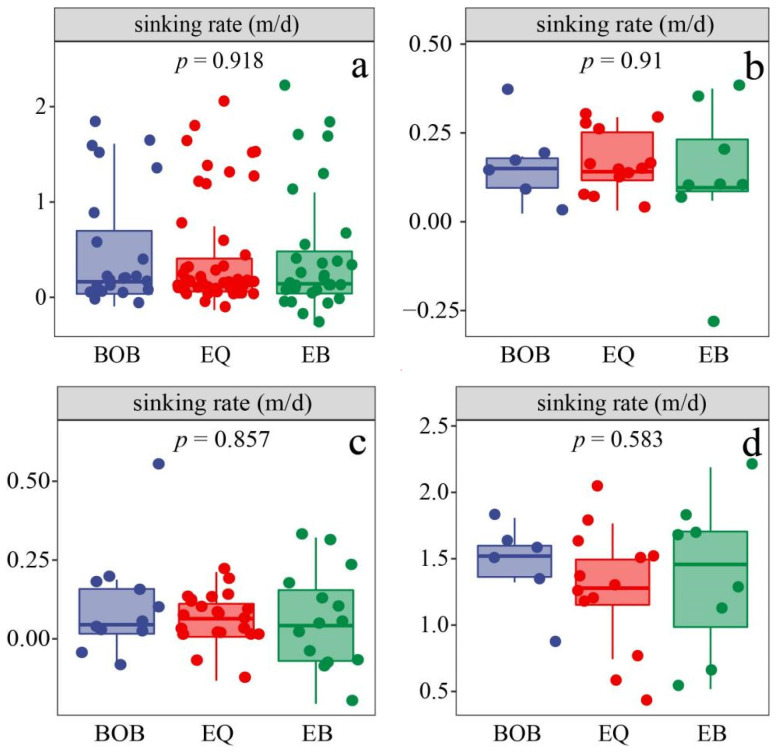
The sinking rates in different sub-regions of the eastern Indian Ocean in spring 2017. (**a**) The average sinking rates in the three sub-regions. (**b**) The average sinking rates of the surface layer (3 m and 25 m) in the three sub-regions. (**c**) The average sinking rates of the middle layer (50 m, 75 m and 100 m) in the three sub-regions. (**d**) The average sinking rates of the bottom layer (150 m and 200 m) in the three sub-regions.

Based on the results of cluster analysis and the vertical variation of sinking rates, we found that the sinking rates in the surface layers (3 m, 25 m) of BOB and EQ were higher than EB (Figure 6b), while the sinking rates in the middle (50 m, 75 m, 100 m) and bottom (150 m, 200 m) of BOB were higher than in the other two regions (Figure 6c,d).

### 3.4. Correlation of Sinking Rates with Biological and Environmental Parameters

**Figure 7:**
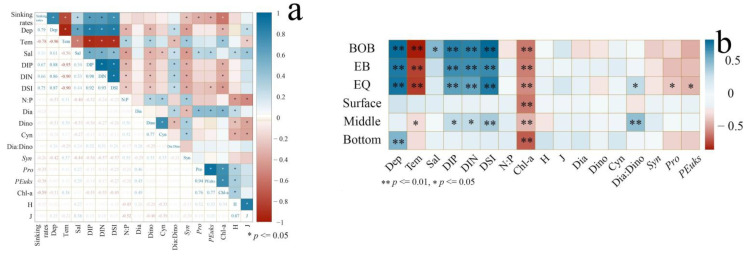
Correlation analysis in the eastern Indian Ocean in spring 2017. (**a**) Relationship between environmental factors, phytoplankton, and sinking rates in the eastern Indian Ocean. (**b**) Correlations between sinking rates and biochemical factors in different sub-regions and at different depths. Pearson correlation coefficients (r) ranged from negative to positive. ** *p* < 0.01; * *p* < 0.05 (two-tailed).

The correlation analysis of phytoplankton sinking rates with biological and environmental factors is shown in Figure 7. The phytoplankton sinking rates were positively correlated with nutrients and depth and negatively correlated with temperature and Chl-*a*. Picophytoplankton were the main variable affecting the phytoplankton sinking rates in the EQ. By comparing the correlation between the sinking rates and phytoplankton and the environmental factors at different depths, the sinking rates in the middle layer were affected by a combination of factors, mainly due to the impact of environmental factors on phytoplankton, and the sinking rate was negatively correlated with DIN, DIP and DSI, and positively correlated with temperature.

**Figure 8:**
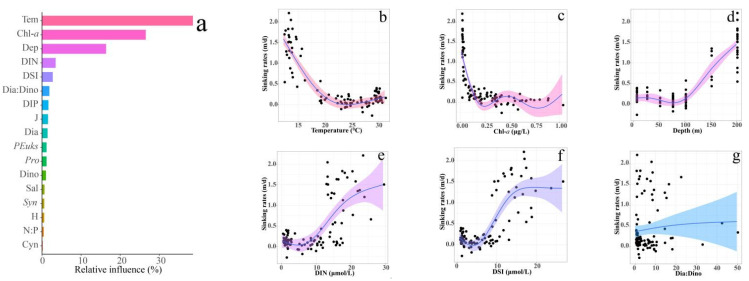
Aggregated boosted tree (ABT) analysis showed the relative effect of sinking rates on Biological factors and chemical factors (**a**) and Results of GAMs describing the sinking rates with temperature (**b**), Chl-*a* (**c**), Depth (**d**), DIN (**e**), DSI (**f**), Dia: Dino (**g**) in the EIO. Solid bule lines represent smoothed mean relationships from GAMs, and shaded areas are 95% confidence intervals. The black dots represent residual values. The model R^2^ value was 0.797.

The top six predictors of the ABT model were nonlinearly fitted using GAMs. The temperature, Chl-*a*, depth, DIN, DSi, and ratio of diatom to dinoflagellates were also strong predictors of the sinking rates in the water column in our GAMs (Figure 8, all *p* < 0.05). The effect of temperature on sinking rates showed a strong correlation with temperature, with a broad peak at 22–23 °C and a subsequent increase. Chl-*a* and sinking rates showed more volatility at 0.25 μg/L, 0.50 μg/L, and 0.75 μg/L. Generally speaking, the sinking rates decrease with the increase in chlorophyll concentration. For depth, DIN, and DIP, there is an inflection point between them and sinking rates. Comparing the vertical distribution of nutrients, it can be seen that they are all near the thermocline of temperature. In addition, the sinking rates increased slowly with an increase in the proportion of diatoms and dinoflagellates.

## 4. Discussion

### 4.1. Coupling of Phytoplankton Sinking Rates with Environmental Factors and Biological

Previous studies have shown that, during the faster sinking rates of phytoplankton, more carbon is transported to the bottom of the ocean. Slowly sinking phytoplankton are easily eaten and decomposed by microorganisms while sinking, and less carbon is transported to the lower layer [2]. Therefore, it is important to recognize the sinking rate of phytoplankton [39]. Theoretical phytoplankton sinking rates are influenced by environmental factors and phytoplankton community structure. GAMs enable us to examine the effects of individual environmental parameters and reveal potential control mechanisms. Our results show that when the temperature is less than 23 ℃, the sinking rates of phytoplankton decrease gradually with the increase in temperature and remain at a low level when the temperature is higher than 23 ℃. For natural sea water, warmer sea water corresponds to lower density, and at the same time, the density of the deep layer is greater than that of the surface layer. Due to lower temperature and increased salinity, the density of the bottom layer is significantly higher than that of the surface layer. These factors will cause the bottom layer to inhibit the sinking of phytoplankton, but our results show the opposite trend. The SETCOL method is performed in the dark; the sinking rate depends on the physiological state of the phytoplankton at the time of sampling. The phytoplankton in the deep layer are senescent cells that settle down from the upper layer and cannot actively regulate the state of their cells, resulting in a higher sedimentation rate. As we all know, DIN and DSI increase with depth, while temperature and illumination decrease with depth. Therefore, we need to comprehensively consider the effects of the opposite factors. In addition, good physiological status is an important factor in phytoplankton’s resistance to sinking. Light and temperature have a great impact on the physiological state of phytoplankton [40]. A large number of indoor experimental results show that the sinking rates of phytoplankton cells are related to their physiological activity. When the cells are in the exponential growth period, the physiological state is the best, and the sinking rates are the slowest; when they are in the declining growth period, their physiological state is the worst, and the sinking rate is the fastest [41,42,43]. Bienfang’s [44] study in subtropical waters found that the sedimentation rate of phytoplankton cells at night was twice that during the day. Many other studies have also found that the sedimentation rate of phytoplankton increases under low sunshine intensity [39,45,46]. The relationship between chlorophyll and sinking rates also shows that phytoplankton cell activity is an important factor in resisting sinking rates. An increase in chlorophyll indicates that the phytoplankton are in the reproduction period, and the sinking rates are generally low. When the chlorophyll concentration reached 1.0 μg/L, the sinking rates increased, which may be related to algal blooms, and Guo’s [27] study showed that algal blooms increase the sedimentation rate of the phytoplankton community.

The ratio of diatoms to dinoflagellates is positively correlated with the sinking rates of phytoplankton in our study, which agreed with a previous investigation reported by Pitcher et al. [10], who found that the sinking rate was lower when dinoflagellates rather than the diatoms were the dominant species. In fact, when diatoms bloom, their sinking rate often increases significantly. Smetacek [47] believed that this represents the transition of diatoms from reproduction to dormancy. Guo et al. [27] found that the sinking rates were higher when the phytoplankton community was dominated by diatoms during summer in the Yangtze River Estuary of China; the sinking rates were low when the phytoplankton community was dominated by dinoflagellates in spring. The correlation analysis of zoning and stratification shows no significant correlation between Pico and sinking rates in each region, which may be due to the vulnerable properties of small phytoplankton to seawater fluctuations.

### 4.2. Characteristics of Phytoplankton Sinking Rate in the Eastern Indian Ocean

The Indian Ocean is adjacent to Asia, and with the change of seasons, the heat exchange between land and sea and the pressure and wind belts also move with the seasons, forming a typical tropical monsoon climate. Furthermore, the BOB receives a large amount of fresh water from the river, and the effect of precipitation is greater than that of evaporation, so the stratification of water is obvious, and the upward transport of nutrients in the lower layer is blocked. The intense and narrow eastward surface currents occurring twice a year within 2° of the equator during the period of monsoon transition in spring and fall are now referred to as Wyrtki Jets (WJs) [48]. The high salinity levels were particularly observed between 80° E and 90° E around the equator. This was probably because the Wyrtki jets was strongest between 60° E and 90° E, and as the water moves, high-salinity water gradually accumulates in the east, resulting in higher salinity and further affecting the structure of the water in the region. At the same time, influenced by upwelling of Sumatra, higher salinity was observed near EB. These factors result in the differences in environmental factors in the investigated regions.

High seawater temperature and low nutrients allowed *Trichodesmium* spp. to grow prosperously during the survey period of the windless season in BOB [49,50,51]. *Trichodesmium* spp. cells contain air bubbles that provide buoyancy for the cells to float on the water surface [52,53,54]. Although the sinking rates of *Trichodesmium* spp. do not accelerate, they cause a further imbalance in the nitrogen and phosphorus ratio [55,56], and the community structure of phytoplankton changes with this process. The rapid growth of phytoplankton leads to faster cell replacement, and the sinking rates of phytoplankton increase [57]. In the middle layer, we find that the sinking rates of BOB and EQ are greater than EB. Combined with the composition of GAMs model and phytoplankton distributions, the increasing diatom in the middle layer is the main factor affecting the sinking rates of phytoplankton [58,59], our results further prove that the increase in diatom abundance increases the sinking rates. In the bottom layer, the sinking rates of BOB are the largest, and those of EQ are the smallest. *Trichodesmium* spp. mainly live on the ocean’s surface, and their presence at the bottom layer means that their own cellular physiological activity can no longer support their resistance to gravity. During the growth process, phytoplankton cells secrete an extracellular mucus of polysaccharide components [60], which is viscous and can adhere phytoplankton cells to each other to form aggregates. The agglomerates are relatively fluffy and filled with a large amount of water, which reduces the average density of phytoplankton agglomerates [61,62] but increases the volume of condensate. According to Stoke’s formula [11], when the effect of density reduction exceeds that of volume increase, it is manifested as a reduction in the phytoplankton sedimentation rate. However, if phytoplankton aggregates adsorb other high-density debris, the average density of aggregates will increase, and the sedimentation rate will increase, which may be the reason for large numbers of *Trichodesmium* spp. at the bottom. In the EIO, seawater stratification caused by temperature changes the distribution of nutrients in the upper layer, and phytoplankton are affected by depth, temperature, and nutrients, resulting in changes in community structure and finally showing different subsidence characteristics.

## 5. Conclusions

The sinking rate of phytoplankton varies among different groups and different species. Here, through the study on the sinking rate of the EIO in 2017, we found that the sinking rate of phytoplankton in an area with high community diversity was significantly lower than that in an area with low community diversity. The correlation between the phytoplankton sinking rate and environmental factors in the three regions is consistent and strong, but the correlation with the phytoplankton community structure is not obvious. However, environmental factors act on phytoplankton cells and affect the whole community structure. Whether from the whole investigated sea area or in the vertical distribution, the appropriate temperature, salinity, and nutrients cause the community structure to reach a stable state (high diversity and evenness) and reach the optimal physiological state of cells, making it easier to resist gravity and stay in the corresponding water layer. By directly measuring the settlement rate of the natural sea area, this study emphasizes the different sinking rates of different communities and the influence of community stability on the sinking rates.

## Figures and Tables

**Table 1:** Dominant species in the eastern Indian Ocean in spring 2017.

Species	*fi*	*pi*	*Y*
*Trichodesmium thiebautii*	91.26	0.312	0.28556
*Prorocentrum compressum*	0.58	0.693	0.00402
*Synedra* spp.	0.53	0.741	0.00393
*Dictyocha fibula*	0.49	0.483	0.00237
*Nitzschia* spp.	0.29	0.714	0.00209
*Prorocentrum leniculatum*	0.38	0.456	0.00172
*Coscinodiscus subtilis*	0.17	0.599	0.00100
*Thalassiothrix longissima*	0.26	0.299	0.00079
*Pyrocystis noctiluca*	0.30	0.252	0.00076
*Oxytoxum* spp.	0.13	0.442	0.00059

*fi* is the frequency of occurrence of species *I* in each sample, *pi* is the probability of cell abundance of species *i* in the samples, *Y* is the dominance index.

## Data Availability

All data are available from the authors upon request.
